# Effect of home-based high-intensity interval training using telerehabilitation among coronary heart disease patients

**DOI:** 10.1097/MD.0000000000023126

**Published:** 2020-11-20

**Authors:** Filip Dosbaba, Martin Hartman, Jakub Hnatiak, Ladislav Batalik, Ondrej Ludka

**Affiliations:** a Department of Rehabilitation, University Hospital Brno; b Faculty of Medicine, Masaryk University, Brno, Czech Republic.

**Keywords:** cardiac rehabilitation, cardiorespiratory fitness, high-intensity interval training, telerehabilitation

## Abstract

**Introduction:**

Cardiovascular diseases are the world's most common causes of morbidity and mortality in the population, including Central Europe. Cardiac rehabilitation (CR) is an effective preventive approach that includes several core components. Physical training is identified as an integral and essential part of CR. Training can positively influence several cardiovascular risk factors in people diagnosed with coronary heart disease and prevent them from clinical events. Our study aims to research the method of high-intensity interval training (HIIT) in a home environment using telerehabilitation. We assume that the HIIT form of telerehabilitation, using a heart rate monitor as a tool for backing up training data, can improve cardiorespiratory fitness and lead to higher peak oxygen uptake than the traditional moderate-intensity continuous training (MICT).

**Methods:**

This study is designed as a monocentral randomized controlled trial at University Hospital Brno in the Czech Republic. After the coronary heart event, the suitable patients will be randomized (1:1 ratio) and separated into 2 groups: the experimental HIIT group and the control MICT group. Both groups undergo a 12-week telerehabilitation with a 1-year follow-up period. Study participants will be telemonitored during physical training in their home environment via a heart rate monitor and a web platform. Once a week, the patients will give their feedback and motivation by a telephone call.

The primary outcome observed will be the effect of intervention expressed by changes in cardiorespiratory fitness. Secondary outcomes will be the health-related quality of life, anxiety, training adherence, body composition, safety, and satisfaction.

**Discussion:**

The HIIT is widely researched predominantly in a center-based supervised form. Our study differs from others by the use of telemedicine and smart technologies in home-based settings. Previous home-based cardiac telerehabilitation studies have focused primarily on MICT, which has demonstrated feasibility, and results have shown similar improvements as center-based CR. There is a presumption that HIIT may be superior to MICT. However, it can be complicated to self-dose the method in the home environment. Investigators expect that HIIT research will provide insight into the possibilities of telemedicine feasibility, effect, and limitations of coronary heart disease patients’ use at low to moderate cardiovascular risk.

## 1 Introduction

Coronary heart diseases (CHD) represent a significant part of all deaths caused by cardiovascular diseases worldwide.^[1]^ CHD mortality prevalence is supposed to increase by 2030 (≈17%).^[2]^ Therefore, it is essential to work on secondary preventative strategies that reduce cardiovascular disease impact. Cardiac rehabilitation (CR) is a complex secondary – preventive intervention that makes an effort to reduce cardiovascular disease risks and to promote, accept, and keep a healthy lifestyle.^[3]^ Secondary prevention recommendations emphasize the importance of receiving a many-sided approach to manage cardiovascular risks.^[4]^ The physical activity represents the essential CR component and plays a vital role in secondary cardiovascular prevention. Systematic exploration of the Cochrane database recorded approximately 12% mortality reduction from all causes and almost 26% of cardiovascular mortality after comprehensive CR program, including physical training.^[5]^ Other meta-analyses support these findings.^[6,7]^ Despite all the recognized benefits, global providing of outpatient CR programs is insufficient.^[8]^ The application of these programs in the Czech Republic is comparable with other world parts. Implementing and completing outpatient programs remains low (≈10%).^[9]^

Considering the main reasons for absence in such programs, patients usually mention technical complications, such as commuting difficulties, time demandingness, or fixed program schedule.^[10]^ Currently, CR focuses on possibilities that might increase patients’ attendance according to their preferences and needs. One of such approaches that shall be explored is using telerehabilitation.

Telerehabilitation involves using information and communication technologies for providing remote health care services.^[11]^ In terms of using CR, telerehabilitation uses information and communication technologies, providing feedback and consultancy from specialists in hospital centers. Telerehabilitation has great potential in improving overall CR uptake.^[12]^

A systematic review of earlier telemedicine CR studies revealed a significant influence of cardiovascular risk factors, including low adherence to the training programs, higher cholesterol, and systolic blood pressure. At the same time, physical fitness values were higher in patients who enrolled in telemedicine groups. Some proofs indicate that telerehabilitation may improve health-related quality of life and decrease CR program costs.^[13]^ Physiological monitoring has been recently identified as an incredibly important trend for future development; nevertheless, its implementation has not yet been adequately managed.^[14]^ Current telemonitoring possibilities are sufficient and enable the analysis of the performed physical training and heart rate (HR). Telecommunication might replace traditional communication tools and implement fast-developing technologies enabling faster and individualized possibilities.^[15]^

A recently completed study demonstrated an intervention using the HR monitor and internet as an effective and sufficient way of providing telerehabilitation in patients after CHD.^[16]^ A 12-week home-based program showed that telerehabilitation leads to similar results in cardiopulmonary fitness as outpatient training. Although this study proved the feasibility and efficiency of telerehabilitation, further randomized control studies are needed in this field.

In the presented research study, we want to verify the inclusion of high-intensity interval training (HIIT) in home-based settings using telerehabilitation. We hypothesize that the HIIT telerehabilitation via HR monitor will improve cardiorespiratory fitness and lead to higher peak oxygen consumption than traditional medium-intensity continuous training (MICT).

## 2 Methods

### 2.1 Study design

**Figure 1:**
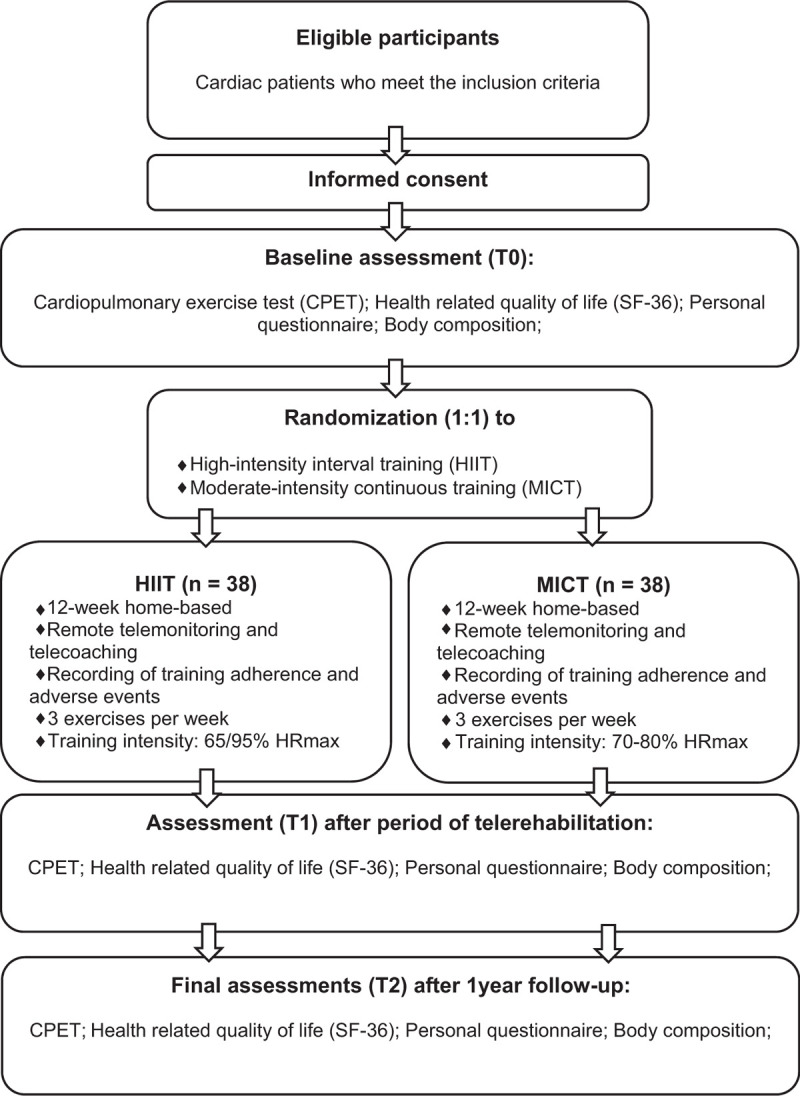
Flowchart of the study protocol.

This study is designed as a monocentral randomized controlled trial at University Hospital (UH) Brno in the Czech Republic. All participants provided written informed consent before entering the study. Study data will be collected at baseline (T0), after 3 months (T1), and after 12 months (T2). (Fig. 1) The Institutional Ethics committee approved the study protocol of the UH Brno, Czech Republic. The trial is registered as recommended by the World Health Organization at ClinicalTrials.gov with number NCT04552652. Study data will be password-protected, and only the designated investigator of the research team can access it. The participants’ data will be processed and backed up following the current European General Data Protection Regulations.

### 2.2 Population and randomization

Participants suitable for this study will be the patients of UH Brno older than 18 years, diagnosed with CHD (angina pectoris or myocardial infraction in last eight weeks), with low or medium cardiovascular risk status (without residual coronary stenosis, value of left ventricular ejection fraction more than 40%).^[17]^ After heart revascularization, the participants will be referred to the II. phase of CR with the recommended pharmacotherapy. Further eligibility criteria will be clinically stable state, ability to perform physical training and cardiopulmonary exercise test (CPET), ability to understand and write in the Czech language, internet connection at home, and literacy with information and communication technology. The participants’ recruitment and allocation will be monitored by the study cardiologist, physiotherapist, or an assistant in UH Brno, Czech Republic.

The exclusion study criteria are patients hospitalized with CHD in the previous 6 weeks, severe psychological or cognitive disorders, serious training limitations besides CHD, participation in another training program under supervision, and contraindications for CPET.

Participants will be randomized (in 1:1 ratio) into the experimental HIIT group and control the MICT group via computer sequence generated by the study statistician. The treatment allocation will be hidden until the completion of the baseline of the initial examinations in sequentially numbered, sealed, opaque envelopes. The study participants cannot be blinded by treatment allocation, but researchers who perform line examinations will be blinded by treatment allocation.

### 2.3 Training program

The exercise program is prescribed according to the current recommendations of the European Society of Cardiology (ESC).^[18]^ In both groups, the patients participate in a 12-week telerehabilitation training program with three training sessions a week. All participants will perform home-based physical training with prescribed exercise intensity according to HR max, determined by the CPET assessment.

**Figure 2:**
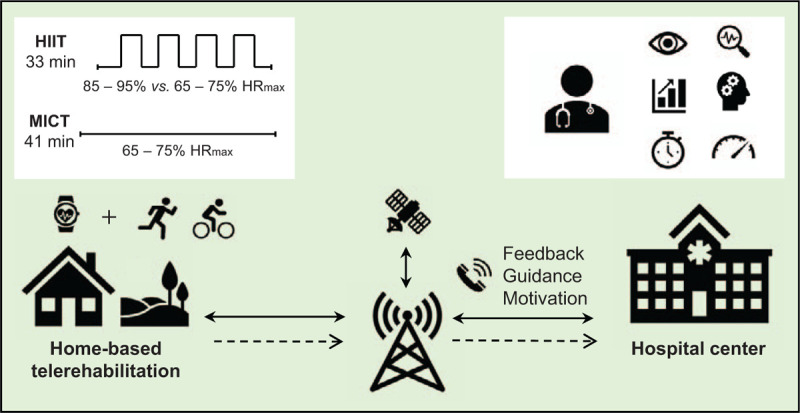
Home-based telerehabilitation scheme. The figure represents home-based physical training via telemonitoring and remote guidance. Icons illustrate cardiac telerehabilitation form. The differences in training prescription between the two study groups are shown in the diagram at the top left.

The prescription of exercise intensity is shown in Figure 2. The HIIT group warms-up for 5 minutes at moderate intensity (65%–75% of HRmax). After warm-up exercise, core training will continue in 4 minutes intervals at high-intensity to reach the target HR zone (85%–95% of HRmax). Each interval will be separated by 3 minutes of active recoveries (at 65%–75% of HRmax). The session ends with a 3-minute cool-down phase. Overall training exercise time will be 33 minutes; isocaloric compared to the MICT group. Patients in the MICT group will perform a 41-minute constant workout with intensity at 65% to 75% of HRmax, representing the same total training load as the HIIT group. All participants will train using an HR monitor during each training session in the home environment.

One to 3 pilot training sessions in the outpatient CR center under the supervision of a physiotherapist will undertake all participants before the telerehabilitation program. These pilot sessions’ objective is to educate and practice how to use a wearable HR sensor (Polar H10). As a part of the education, the patients learn how to adhere to the prescribed duration of the physical training and intensity by checking the correct HR zone. Further on, the patients will be instructed about preferred training modality in a home-based environment (cycling, walking, or Nordic walking) and receive advice on how to perform the modality. Subsequently, the patients will be educated on how to use the HR sensor correctly and upload the training data to the web application (Polar Flow).

Throughout the study, the patients will be able to check training data and results to see if they are meeting their training goals. The personal coach (physiotherapist) will be able to view the data in the application and also provide remote guidance (motivation, feedback on the frequency, duration, and intensity of training) via a preferred phone call once a week (Fig. 2). After 3 months, the telemonitored training sessions will be completed. Patients will be recommended to keep on doing exercises with the HR sensor and web application. A long-term effect will be assessed in a 1-year follow-up.

### 2.4 Telemonitoring

The personal coach will keep telemonitoring participants – checking their physical intervention and provide them with feedback and support. At the beginning of the training program, the personal coach will meet each patient separately and set personal training goals.

Such an approach can positively influence the overall improvement because it concretizes relations and enables 1 to individualize each patient's possibilities.

The activity and cooperation will be supported to make the patient feel responsible for their results. Remote telemonitoring and supervision will meet the following criteria: The training program will be set into three parts. The golden standard for CR recommends the warm-up phase, core exercise, and cooling-down phase. Each phase will take the exact time and be set training intensity according to HRmax assessed in CPET. The coach will monitor whether the patient keeps the stages and the training HR intensity. Based on the analysis, the coach will make some changes or provide recommendations for the program's rest.

### 2.5 Outcomes

The primary study outcome is the cardiorespiratory fitness value, assessed at the beginning of the intervention and after a 12-week and 1-year follow-up period. The study's secondary intervention outcome is the health-related quality of life, anxiety and depression, body composition, the incidence of treatment-emergent adverse events assessed by 5-grade scale, training adherence (number of compliant participants, average exercise time, overall completition rate). Assessment of the cardiorespiratory fitness, health-related quality of life, depression, anxiety, and body composition will be evaluated at the beginning of the study (T0), after 12-weeks (T1), and after 1-year period (T2).

### 2.6 Cardiorespiratory fitness

Analysis and assessment of cardiorespiratory fitness will be processed via automated metabolic system Metalyzer 3b (Biophysics GmbH, Leipzig, Germany) and by the progressive increment CPET on bicycle ergometer Ergoselect 100 (Ergoline, Bitz, Germany) up to the level of limited symptom maximum. CPET will be set according to recommendations of ESC and the American Heart Association.^[19,20]^

The study will use ramp protocol for a stress test, including 2 minutes warming up at 10 watts, followed by 15 watts growths every minute for men and 10 watts growths for women with the final 2 minutes of cooling down at 10 watts.

Considering the maximum oxygen consumption is very difficult to reach in CHD patients, it is common to use peak assessed during the stress test as the average in the last 30 seconds of stress.^[20]^ The cardiologist will be present while testing the participants, estimates the abnormal signs on ECG, and independently values ventilation thresholds.

### 2.7 Health-related quality of life

Health-related quality of life will be assessed in the standardized questionnaire SF-36. The questionnaire version has been translated into the Czech language.^[21]^ Normative representative study validity, reliability, sensitivity, and task complexity for the European population has been proven.^[22]^ SF-36 was chosen in our study as an assessment tool for its full usability, functional coherence, and high responsibility in repeated testing. It is very often used in studies focusing on CR.^[23]^

### 2.8 Training adherence and adverse events

Training adherence and incidence of the adverse events will be recorded in the exercise datasheets from the telemonitoring platform for both groups during the 12-weeks training program.

According to the number of completed training sessions, training adherence will be evaluated through the Polar Flow web application as the overall completion rate of prescribed exercise lessons (100% = 36). The training diary in the web application will provide information about the total average time of all exercises in the defined HR zone and the total number of compliant participants (the criterion of compliance with the rehabilitation program is set at 70% of the prescribed training sections). According to the data obtained, investigators will assess the total training program completion.

The study participants will be encouraged to use the contact details for reporting the incidence of treatment-emergent adverse events at any time throughout the study period. The scale will assess the type, incidence, and severity of adverse events (grade 1: mild, asymptomatic or mild symptoms; grade 2: moderate, minimal; grade 3: severe or medically significant; grade 4: life-threatening consequences; and grade 5: death) will be noted.

### 2.9 Anxiety and depression

The hospital anxiety and depression scale will be assessed in the study period from baseline to 12 weeks and 1 year. The questionnaire comprises 7 questions for anxiety and 7 questions for depression. Each item in the questionnaire is scored from 0 to 3 and which means every person can score between 0 and 21 for either anxiety or depression. The hospital anxiety and depression scale is a validated measure licensed to CR to assess anxiety and depression symptoms and is recommended for use with patients with CHD.^[24]^

### 2.10 Body composition

Anthropometrics of the study patients will be assessed at all three-time points (T0–T3). Bodyweight (accuracy = 0.1 kg) and body composition, including total muscle mass, fat mass, and body water, will be measured by bioelectrical impedance analysis from analyzer InBody 370S (BridgePower Corp, Suwon, Korea). Bioelectrical impedance analysis is a non-invasive rapid examination for evaluating body composition.^[25,26]^

Body mass index will be calculated through a simple calculation using a patient's height and weight. The formula is Body mass index = kg / m2, where kg is a patient's weight in kilograms, and m2 is the patient's height in meters squared.

### 2.11 Statistics

The Shapiro-Wilk test will be used to evaluate normality. Comparisons between subjects will be made using the Student *t* test and Mann–Whitney *U* test. The 2-tailed Fisher exact test will test differences in proportions. Data will be summarized as a mean ± standard deviation; *P* values < .05 will be considered statistically significant. Statistica software 12.0 (TIBCO Software Inc, Palo Alto) will be used for statistical analysis.

### 2.12 Power analysis

The group size is based on an anticipated increase of cardiorespiratory fitness 2.8 mL/kg/min with a standard deviation of 4.3 mL/kg/min from the previous study.^[16]^ Seventy-six participants will need to be included in the study to achieve 80% statistical power at 5% statistical significance. The drop-out rate in follow-up at 12 week and follow-up is taken into account, that is, 38 participants need to be randomized to the experimental and control arm of the study.

## 3 Discussion

Patients with CHD usually show a significant improvement in cardiorespiratory fitness and health-related quality of life associated with physical training. Improvements in fitness levels have been described in central-based CR programs and, more recently, in home-based settings.^[27,28]^

The traditionally represented method of physical training in studies is MICT. An alternative to MICT in CR has recently been implementing the HIIT training method that provides a more significant improvement in cardiorespiratory fitness values and takes less time, leading to better clinical outcomes in the CHD population.^[29,30]^ On the other hand, the use of the HIIT method at home-based environment can be more difficult and less accurate, as specialists can more precisely adjust the required training intensity in CR clinical centers as needed.

Telemonitoring and telecoaching are also unique and excellent features of telerehabilitation training that facilitate more controlled physical activity. Besides, they allow remote and electronic monitoring of subjects’ compliance with the training, which is a vital and essential characteristic of the approach, which could reliably assess compliance with the training protocol.

Nevertheless, it is crucial to understand this method, which appears to be a better alternative. Scientific proclamation of the American Heart Association recommends focusing on HIIT and assess its impact and safety.^[12]^

The option of telerehabilitation is significant for patients with CHD who cannot (or do not want to) participate in traditional rehabilitation programs. Recent meta-analyze have shown that telerehabilitation technologies are of great importance in preventing CHD, as they can improve the cardiovascular risk factors, functional capacity, and quality of life of those treated remotely.^[31]^ ESC has fully supported the development and expansion of domestic CR.^[32,33]^

This study's conclusion and the main goal will be to fully evaluate HIIT's usefulness as a method of home training in patients with CHD in comparing the effectiveness of the training approach with the results of the traditional MICT protocol. We anticipate that as home-based rehabilitation therapy, HIIT will be superior to standard traditional MICT in improving cardiorespiratory fitness in CHD.

If we show that HIIT significantly increases cardiorespiratory parameters, it means better results in the CHD population, leading to a decrease in mortality and hospital readmission reduction.^[6]^

Secondary analyzes may suggest that even shorter CR programs may lead to better long-term results. If we can prove that telerehabilitation is a safe intervention, it can mean a step towards implementing this method into standard preventive care. The importance of safety and control of home CR remains a challenge, especially as the population of CHD patients ages and the number of comorbidities and the severity of cardiovascular risk increase.^[34]^ The telerehabilitation approach has the potential to positively influence barriers and improve the overall use of the CR program.

## Author contributions

**Conceptualization:** Filip Dosbaba, Martin Hartman.

**Funding acquisition:** Ladislav Batalik.

**Investigation:** Filip Dosbaba.

**Methodology:** Filip Dosbaba, Martin Hartman, Jakub Hnatiak.

**Project administration:** Filip Dosbaba, Martin Hartman.

**Supervision:** Ondrej Ludka.

**Validation:** Ondrej Ludka.

**Visualization:** Jakub Hnatiak, Ladislav Batalik.

**Writing – original draft:** Filip Dosbaba, Jakub Hnatiak.

**Writing – review & editing:** Ladislav Batalik, Ondrej Ludka.
